# Spirocyclic dimer SpiD7 activates the unfolded protein response to selectively inhibit growth and induce apoptosis of cancer cells

**DOI:** 10.1016/j.jbc.2022.101890

**Published:** 2022-04-01

**Authors:** Smit Kour, Sandeep Rana, Sydney P. Kubica, Smitha Kizhake, Mudassier Ahmad, Catalina Muñoz-Trujillo, David Klinkebiel, Sarbjit Singh, Jayapal Reddy Mallareddy, Surabhi Chandra, Nicholas T. Woods, Adam R. Karpf, Amarnath Natarajan

**Affiliations:** 1 Eppley Institute for Research in Cancer and Allied Diseases, Fred & Pamela Buffett Cancer Center, University of Nebraska Medical Center, Omaha, Nebraska, USA; 2 Department of Biology, University of Nebraska-Kearney, Kearney, Nebraska, USA

**Keywords:** small molecule, isatin-derived spirocyclic dimer, protein misfolding, UPR, UPR activation, cancer cell vulnerability, apoptosis, ATF6, activating transcription factor 6, BiP, binding immunoglobulin protein, CHOP, C/EBP homologous protein, eIF2α, eukaryotic initiation factor 2α, EIF2AK3, eukaryotic translation initiation factor 2 alpha kinase 3, ER, endoplasmic reticulum, ERAD, ER-associated degradation, HGSC, high-grade serous carcinoma, IKKβ, inhibitor of NF-κB kinase subunit beta, IRE1, inositol-requiring enzyme 1, MS, mass spectrometry, PARP, poly(ADP-ribose) polymerase, PERK, protein kinase R–like ER kinase, pS51, phosphorylation of serine 51, RELA, v-rel avian reticuloendotheliosis viral oncogene homolog A, SEC, surface-exposed cysteine, Tg, thapsigargin, TPE-MI, tetraphenylethene maleimide, UNMC, University of Nebraska Medical Center, UPR, unfolded protein response, XBP1, X-box binding protein 1

## Abstract

The unfolded protein response (UPR) is an adaptation mechanism activated to resolve transient accumulation of unfolded/misfolded proteins in the endoplasmic reticulum. Failure to resolve the transient accumulation of such proteins results in UPR-mediated programmed cell death. Loss of tumor suppressor gene or oncogene addiction in cancer cells can result in sustained higher basal UPR levels; however, it is not clear if these higher basal UPR levels in cancer cells can be exploited as a therapeutic strategy. We hypothesized that covalent modification of surface-exposed cysteine (SEC) residues could simulate unfolded/misfolded proteins to activate the UPR, and that higher basal UPR levels in cancer cells would provide the necessary therapeutic window. To test this hypothesis, here we synthesized analogs that can covalently modify multiple SEC residues and evaluated them as UPR activators. We identified a spirocyclic dimer, SpiD7, and evaluated its effects on UPR activation signals, that is, *XBP1* splicing, phosphorylation of eIF2α, and a decrease in ATF 6 levels, in normal and cancer cells, which were further confirmed by RNA-Seq analyses. We found that SpiD7 selectively induced caspase-mediated apoptosis in cancer cells, whereas normal cells exhibited robust *XBP1* splicing, indicating adaptation to stress. Furthermore, SpiD7 inhibited the growth of high-grade serous carcinoma cell lines ~3-15-fold more potently than immortalized fallopian tube epithelial (paired normal control) cells and reduced clonogenic growth of high-grade serous carcinoma cell lines. Our results suggest that induction of the UPR by covalent modification of SEC residues represents a cancer cell vulnerability and can be exploited to discover novel therapeutics.

Unfolded protein response (UPR) is an adaptation mechanism designed to handle increased folding needs in cells and restore proteostasis. In normal cells, extrinsic stressors such as nutrient deprivation or acidosis trigger transient activation of UPR to restore proteostasis. Failure to restore proteostasis activates an apoptosis program. UPR is a protective mechanism designed to handle stress, and the level/intensity of stress dictates cell fate decisions (1, 2, 3, 4, 5, 6, 7, 8). Under acute endoplasmic reticulum (ER) stress conditions, adaptation mechanisms, such as expression of chaperones to increase folding capacity and ER-associated degradation (ERAD) proteins to clear misfolded proteins, are activated to restore normal homeostasis (9, 10, 11). However, under chronic ER stress, the adaptation mechanisms are overwhelmed, and cell death pathways are activated by, among others, transcription factor C/EBP homologous protein (CHOP) (12). In addition to cell extrinsic stressors that are transiently experienced by normal cells, tumor cells also face cell intrinsic stressors such as loss of tumor suppressors and oncogene addiction. A recent study showed that loss of the tumor suppressor phosphatase and tensin homolog deleted on chromosome 10 in high-grade serous carcinoma (HGSC) was associated with a higher burden of misfolded proteins. Phosphatase and tensin homolog deleted on chromosome 10 loss resulted in (a) higher basal levels of UPR-associated proteins and (b) increased sensitivity to bortezomib (13). This higher basal proteostasis makes cancer cells particularly vulnerable to UPR activation–induced apoptosis. Figure 1 is a model for the aforementioned, wherein the resting proteostasis in cancer and normal cells is shown in *red* and *green*, respectively. The effect of UPR activation is shown as *red* and *green arrows* in cancer and normal cells, respectively. The therapeutic window shown as a double-headed arrow, that is, the difference between the resting proteostasis levels of cancer cells and normal cells is the same for UPR activators and UPR inhibitors. Theoretically, from a therapeutic standpoint, UPR activators will drive cancer cells toward apoptosis, whereas UPR inhibitors will reduce the proliferative capacity of the cancer cells.

**Figure 1:**
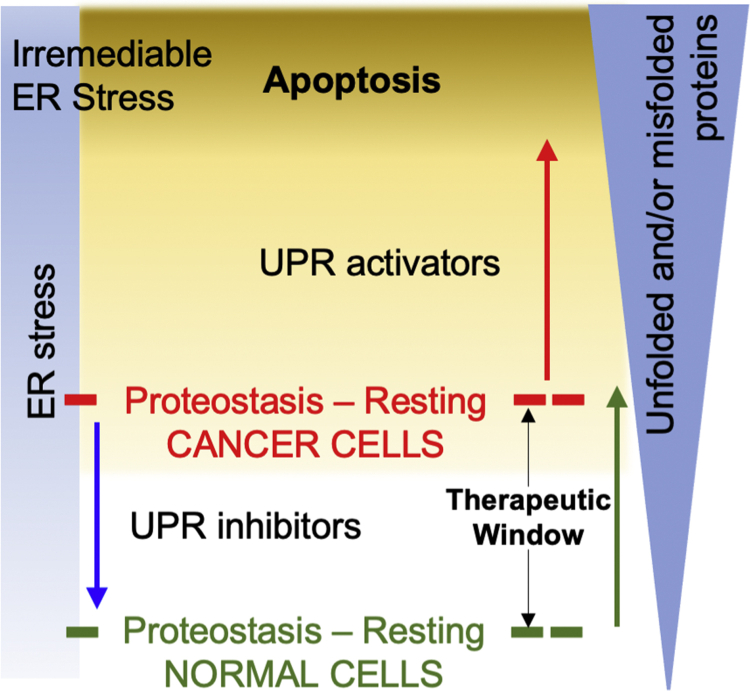
**Rationale for the development of UPR inhibitors and UPR activators.** The *red* and *green dotted lines* indicate hypothetical resting proteostasis levels in cancer and normal cells, respectively. The *red* and *green arrows* indicate UPR activation in cancer and normal cells. The *blue arrow* indicates the effects of UPR inhibition in cancer cells. UPR, unfolded protein response.

UPR activation is regulated by three major pathways, namely activating transcription factor 6 (ATF6), protein kinase R–like ER kinase (PERK), and inositol-requiring enzyme 1 (IRE1). Although all three pathways upregulate the expression of chaperones, IRE1 and PERK pathways also upregulate ERAD and apoptosis-associated genes, respectively. Activation of UPR pathways is monitored by specific readouts. Decrease in ATF6 levels serves as a sensor for ATF6 pathway activation. Thapsigargin (Tg)-induced ER Ca^2+^ depletion leads to the proteolytic cleavage of membrane-bound ATF6 to release soluble ATF6(p50), which translocates to the nucleus and activates transcription of the chaperone binding immunoglobulin protein (BiP) (11). Hypoxia-induced stress leads to the activation of PERK pathway. This is indicated by hyperphosphorylation of PERK and phosphorylation of serine 51 (pS^51^) on eukaryotic initiation factor 2α (eIF2α) (14). These phosphorylation events serve as readouts for PERK pathway activation. Increased level of unfolded proteins causes IRE1-dependent splicing of a small intron from X-box binding protein 1 (XBP1) (15, 16, 17). Spliced XBP1 is a sensor for the IRE1–XBP1 pathway that transcriptionally regulates levels of ERAD components (18).

A wide array of small molecules that directly or indirectly perturb UPR have been reported (19). The therapeutic window, that is, the difference in resting proteostasis in cancer cells *versus* normal cells, suggests that both UPR inhibitors and UPR activators can be explored as cancer therapeutics (Fig. 1). Examples include BiP inhibitor YUM70 that exhibits *in vitro* and *in vivo* effects in pancreatic cancer models (20, 21). Studies with IRE1 inhibitor MKC8866 in multiple prostate cancer models revealed that it targeted the prosurvival role of IRE1–XBP1 axis that activated c-Myc (22, 23). Knockout and mutational studies with PERK and eIF2α^S51A^, respectively, impairs tumor growth in hypoxic regions because of impaired integrated stress response (24). PERK inhibitors, GSK2606414 and GSK2656157, exhibited antitumor effects; however, pancreatic damage because of dose-limiting toxicity remains a concern (25, 26, 27). Along with PERK, general control nonderepressible 2 and heme-regulated inhibitory kinase are eIF2α kinases that are known to elicit integrated stress response. An example of a direct activator of UPR is the urea analog (3r) that inhibited tumor growth by heme-regulated inhibitory kinase–mediated phosphorylation of eIF2α (28). UPR activators that do not directly target the proteins associated with UPR have also been identified. Compound 147, identified through an elegant screen, covalently modifies protein disulfide isomerases to preferentially activate ATF6 (29). Proteasome inhibitors (Velcade, Ninlaro, and Kyprolis) elevate the levels of ubiquitinated proteins, thereby activating UPR-mediated cell death. A characteristic of misfolded proteins is a higher percentage of surface-exposed hydrophobic patches. This is recognized by the endogenous protein quality control machinery and activates their degradation. This inspired several laboratories to append hydrophobic tags to high-affinity small molecules to drive the degradation of the target proteins (30, 31, 32, 33, 34, 35, 36, 37, 38). Along these lines, we explored covalent modification of surface-exposed cysteine (SEC) residues to simulate elevated levels of unfolded/misfolded proteins to selectively induce UPR-mediated cancer cell death.

## Results

### Analog **19** covalently modifies >330 proteins

**Figure 2:**
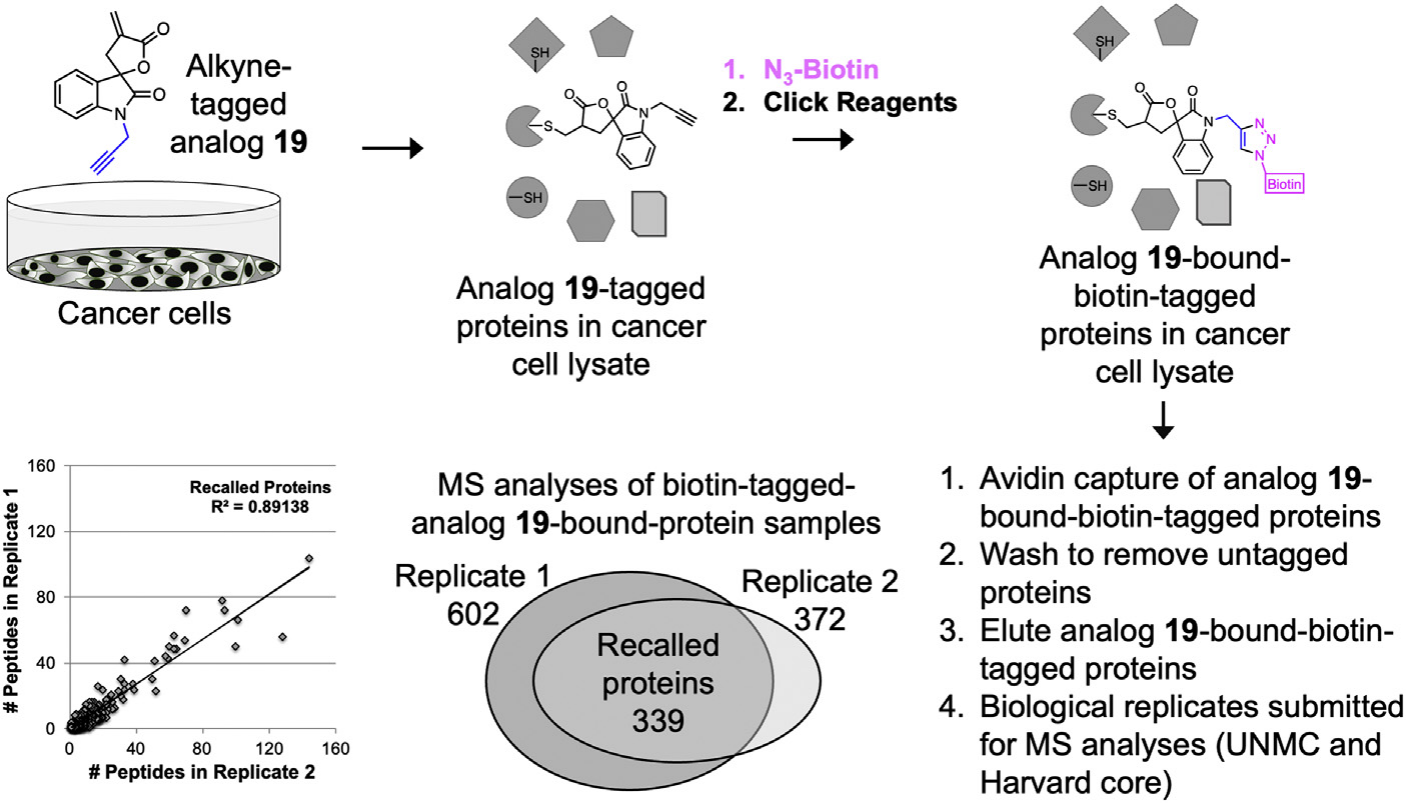
**Identification of proteome-wide targets of 19 in MiaPaCa2 cells.** Cells were incubated with 10 μM of alkyne-tagged monomer **19** for 1 h. The lysates were subjected to click reagents and biotin-azide. Biotinylated **19**-tagged proteins were isolated on a streptavidin column. Following elution proteins were subjected to mass spectrometry analyses. Venn diagram showing the recalled number of proteins identified in the biological replicates. A scatter plot of the number of peptides in the 339 recalled proteins from the biological replicates.

We previously reported the discovery of a spirocyclic compound with an α-methylene-γ-butyrolactone (**19**) that covalently modified NF-κB pathway proteins, RELA (v-rel avian reticuloendotheliosis viral oncogene homolog A) and IKKβ (inhibitor of NF-κB kinase subunit beta), by targeting SECs (39, 40, 41). To identify proteome-wide targets of analog **19**, we conducted a click-mass spectrometry (MS) study using an alkyne-tagged analog **19** (Fig. 2). A total of 635 proteins were covalently bound by analog **19** (peptide threshold >95%; protein threshold >99% with one peptide minimum) from two runs (Table S1). RELA and IKKβ are low abundant proteins, and they were not identified in the click-MS study. The biological replicates recalled ∼53% (339) of proteins that were bound to analog **19**, and 6% of the recalled proteins are localized to the ER (42). Pathway analysis (reactome, version 79; reactome.org) revealed that 14 of 20 most significant pathways were related to translation of proteins (Table S2). Since analog **19** covalently modifies >330 proteins, we hypothesized that a dimer of analog **19** with two covalent modifiers would either covalently modify multiple residues in these proteins to mimic a large hydrophobic patch on the surface or bind to proximal Cys residues on two proteins to simulate a misfolded protein and activate UPR.

### Dimer of analog **19** (SpiD7) activates UPR

**Figure 3:**
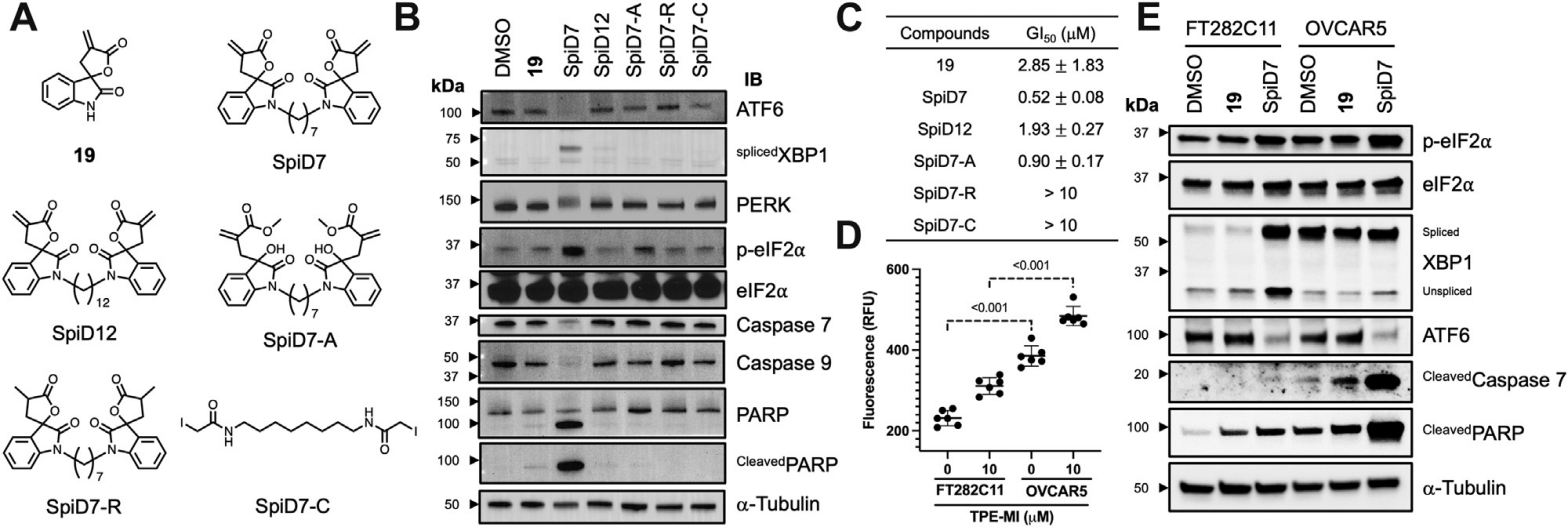
**Effect of SpiD7 and its analogs on UPR-associated proteins, cancer cell growth, and apoptosis.***A*, structure of compounds used for the structure–activity relationship (SAR) study. *B*, cancer cells (OVCAR5) treated with 10 μM of **19**, SpiD7, SpiD12, SpiD7-A, SpiD7-R, or SpiD7-C and incubated for 6 h. The lysates were probed for the indicated UPR and apoptosis-associated proteins. *C*, cancer cells (OVCAR5) treated with varying concentrations of **19**, SpiD7, SpiD12, SpiD7-A, SpiD7-R, or SpiD7-C and incubated for 72 h, and growth inhibition (GI) was determined using Alamar Blue (n = 3, average ± SD). *D*, immortalized fallopian tube epithelial cells (FT282C11) and cancer cells (OVCAR5) were incubated with 10 μM of fluorescent dye TPE-MI, and fluorescence was recorded after a 10 min incubation (n = 6, average ± SD, *p* value two-tailed *t* test). *E*, immortalized fallopian tube cells (FT282C11) and cancer cells (OVCAR5) were treated with 10 μM of **19** and SpiD7 and incubated for 2 h. The lysates were probed for the indicated UPR and apoptosis-associated proteins. Blots shown are representative examples of at least biological replicates. UPR, unfolded protein response.

To test this hypothesis, we assembled dimers of analog **19** with 7-carbon and 12-carbon linkers (SpiD7 and SpiD12) along with an acyclic dimer (SpiD7-A), dimer without the covalent modifier (SpiD7-R), and a diiodo compound (SpiD7-C) and evaluated them for UPR activation. Briefly, OVCAR5 cells were incubated with the aforementioned compounds (Fig. 3*A*), and the lysates were probed for proteins in the UPR pathways (Fig. 3*B*). The results showed that SpiD7 was the only compound that reduced full-length ATF6 levels which is indicative of cleavage-induced activation and induced XBP1 splicing. We also observed PERK activation (the slower moving p-PERK band) resulting in eIF2α phosphorylation. Moreover, in SpiD7-treated lysates, we observed reduced caspase 7 and 9 levels, along with increased levels of cleaved poly(ADP-ribose) polymerase (PARP). We did not observe any such effects in **19**, SpiD12, SpiD7-A, SpiD7-R, and SpiD7-C indicating that the (a) linker length, (b) spirocyclic core, and (c) Michael acceptor contribute to UPR activation and induction of apoptosis. Consistently, results from a growth inhibition assay showed that SpiD7 was the most potent among the panel of analogs screened (Fig. 3*C*).

### SpiD7 activates UPR in both normal and cancer cells and selectively induces apoptosis in cancer cells

The levels of SEC residues are greater in unfolded proteins as compared with their folded counterparts. A thiol probe tetraphenylethene maleimide (TPE-MI) was used to measure unfolded protein levels in cells by binding to free sulfhydryl groups on cysteine residues present on unfolded proteins, which are otherwise buried in their folded counterparts (43, 44). We treated immortalized fallopian tube epithelial cells (FT282C11) (45, 46), which represent a normal cell control, and cancer cell lines (OVCAR5 and OVCAR8) with TPE-MI to assess relative levels of unfolded proteins. We observed high TPE-MI fluorescence in cancer cells suggesting elevated levels of SECs (unfolded proteins) (Figs. 3*D* and S1).

Next, we treated FT282C11 and OVCAR5 cells with **19** or SpiD7 to determine their effect on UPR activation. In dimethyl sulfoxide–treated samples, we observed elevated levels of ^spliced^XBP1 in OVCAR5 cells when compared with FT282C11 cells (Fig. 3*E*, lanes 1 *versus* 4). This suggests that UPR is activated (higher resting proteostasis, Fig. 1) in resting cancer cells compared with normal cells. Analog **19** did not affect the levels of pS^51^-eIF2α, XBP1 splicing, or ATF6, demonstrating that **19** does not activate UPR in either OVCAR5 or FT282C11 cells (Fig. 3*E*, lanes 1 *versus* 2 and 4 *versus* 5). In SpiD7-treated FT282C11 (Fig. 3*E*, lanes 1 *versus* 3) and OVCAR5 (Fig. 3*E*, lanes 4 *versus* 6) cells, we observed an increase in pS^51^-eIF2α levels. A robust increase in XBP1 levels was observed in SpiD7-treated FT282C11, whereas no such increase was observed in OVCAR5 cells (Fig. 3*E*, lanes 1 and 3 *versus* 4 and 6). SpiD7 nonselectively activated ATF6 (Fig. 3*E*, lanes 3 and 6), which is consistent with reported small-molecule probes that covalently target cysteine residues on protein disulfide isomerases to activate ATF6 (29). Moreover, we observed a more robust ^Cleaved^Caspase 7 and ^Cleaved^PARP levels in SpiD7-treated OVCAR5 cells when compared with FT282C11 cells (Fig. 3*E*, lanes 3 *versus* 6). We also compared SpiD7 and the classical UPR activator Tg for induction of apoptosis in normal and cancer cells (Fig. S2). Unlike Tg, SpiD7-mediated PARP cleavage is approximately 2.7-fold higher in OVCAR5 cells as compared with FT282C11 cells.

### RNA-Seq analyses indicate that SpiD7 activates UPR

**Figure 4:**
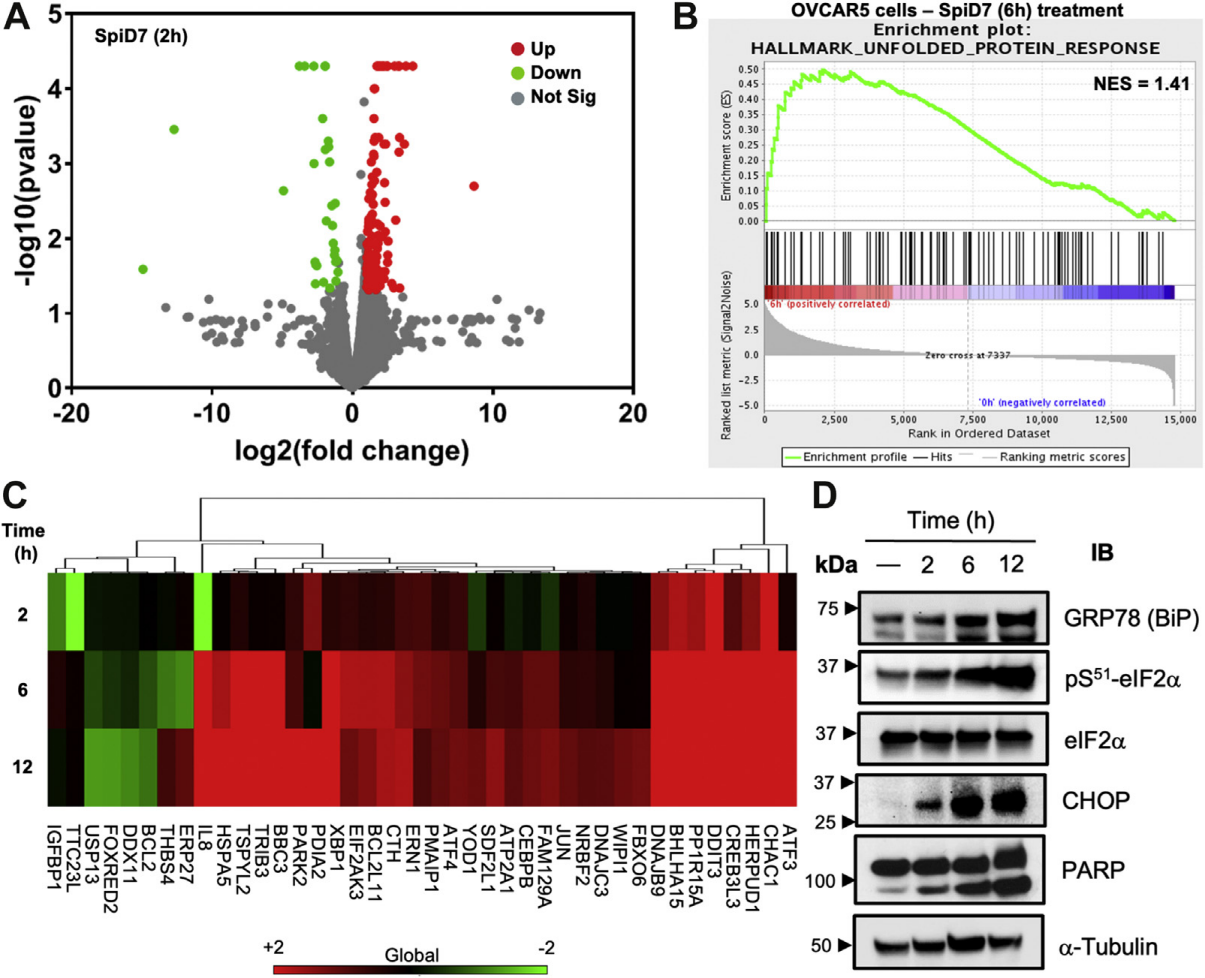
**RNA-Seq analyses with samples derived from cancer cells (OVCAR5) treated with SpiD7 (10 μM) for 2, 6, and 12 h.***A*, representative volcano plot showing the differential RNA expression between the 2 h vehicle and SpiD7 treatment against *p* value (≤0.05 and twofold change is highlighted in *red* and *green*). *B*, representative gene set enrichment analysis for the hallmark UPR gene set for 6 h, SpiD7-treated cells (FDR ≤ 0.05). *C*, heat map showing twofold change of the GO ER stress gene set for the 2, 6, and 12 h, SpiD7-treated cells. *D*, Western blot analyses of lysates derived from cancer cells (OVCAR5) treated with SpiD7 (10 μM) for 2, 6, and 12 h probed for key proteins that indicated UPR activation. Blots shown are representative examples of at least a biological replicate. ER, endoplasmic reticulum; FDR, false discovery rate; GO, Gene Ontology; UPR, unfolded protein response.

To further assess if SpiD7 activates UPR, OVCAR5 cells were treated with SpiD7 and incubated for 2, 6, and 12 h. RNA isolated from these samples was sequenced by the University of Nebraska Medical Center (UNMC) sequencing core. Representative volcano plot describing differential RNA expression between vehicle-treated and SpiD7-treated cancer cells for 2 h is shown in Figure 4*A* (please see Fig. S3 for the volcano plot for the 6 and 12 time points). A greater than two-fold gene expression change was observed in >113 genes at a false discovery of rate <0.05. Gene set enrichment analysis showed a time-dependent increase in the normalized enrichment scores for the hallmark UPR gene set (Fig. 4*B*, 6 h treatment and Fig. S3 for the 2 and 12 h treatment). Euclidean cluster analysis greater than twofold change for the Gene Oontology ER stress and hallmark UPR gene set for 2, 6, and 12 h treatment is summarized in Figure 4*C* and Fig. S4, respectively. A time-dependent increase in the expression for majority of genes (ATF3, HERPUD1, CHAC1, DNAJC3, WIPI1, CEBPB, DDIT4, ERN1, eukaryotic translation initiation factor 2 alpha kinase 3 [EIF2AK3], ATF4, DNAJB9, XBP1, heat shock protein family A member 5 [HSPA5], and TSPYL2) in the Gene Ontology ER stress and the hallmark UPR gene set was observed demonstrating induction of UPR. Of note, overexpression of genes CHAC1, ATF3, ATF4, and EIF2AK3 are associated with proapoptotic effects mediated by the PERK/ATF4/CHOP signaling pathway. Western blot analyses with lysates of the time-course study done in parallel revealed a time-dependent increase of BiP (GRP78) and pS^51^-eIF2α in SpiD7-treated cells (Fig. 4*D*). The time-dependent increase in CHOP and PARP cleavage suggests inability of the cancer cells to overcome the SpiD7-induced covalent modification of proteins (Fig. 4*D*).

### Efficacy studies with SpiD7

**Figure 5:**
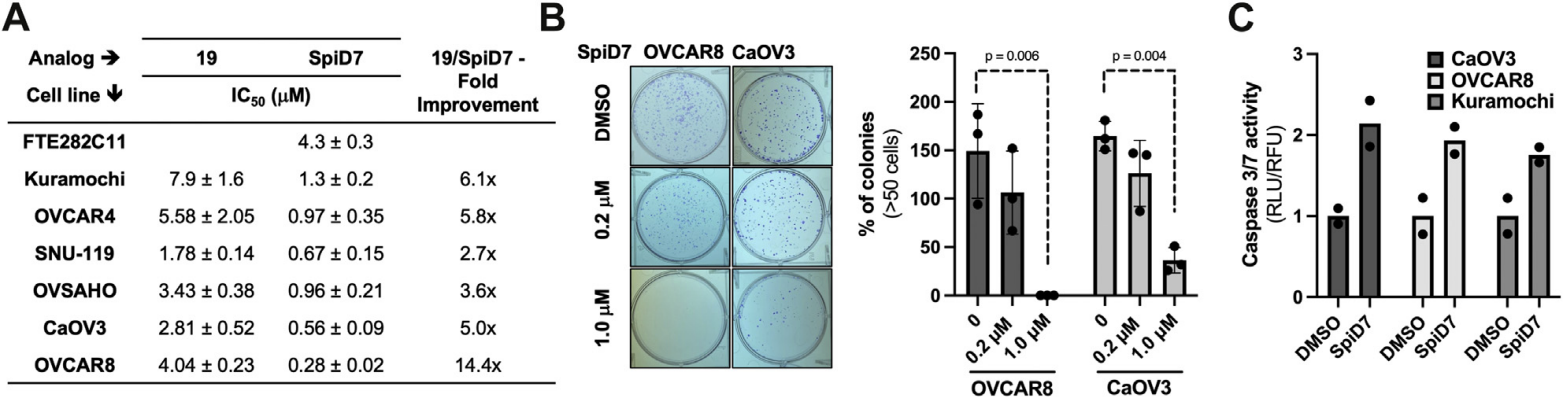
**Efficacy studies with SpiD7 in HGSC cell lines.***A*, growth inhibition (3 days) assay with monomer **19** and dimer SpiD7 in a panel of HGSC cell lines (n = 3, average ± SD). *B*, clonogenic survival studies with dimer SpiD7 in HGSC cell lines. The *bar graph* represents quantification of the number of colonies with more than 50 cells (n = 3, average ± SD, *p* value two-tailed *t* test). *C*, under multiplexing conditions, HGSC cell lines were incubated with SpiD7 for 24 h and caspase 3/7 activity assessed (n = 2). HGSC, high-grade serous carcinoma.

Next, to determine the relative potency of the SpiD7 and **19** to inhibit growth of HGSC cell lines (47, 48), we subjected them to a 3-day cell growth assay. Among the cell lines (Kuramochi, OVCAR4, SNU-119, OVSAHO, CaOV3, and OVCAR8), the most sensitive cell line was OVCAR8, and SpiD7 was 14.4-fold more potent than **19**, and the least sensitive cell line was Kuramochi. On an average, SpiD7 was approximately sixfold more potent than **19** in inhibiting HGSC cell growth. SpiD7 was approximately 3- to 15-fold more potent in inhibiting the growth of HGSC cells over FT282C11 cells (Fig. 5*A*). To assess the ability of SpiD7 to inhibit colony formation, OVCAR8 and CaOV3 cells were subjected to 7- and 14-day clonogenic assay, respectively (Fig. 5*B*). Briefly, single-cell suspension of ∼10^3^ HGSC cells was incubated with SpiD7, colonies allowed to form for 7 or 14 days, stained with crystal violet, and colonies containing over 50 cells were manually counted. SpiD7 reduced clonogenic growth and survival of HGSC cells (OVCAR8 and CaOV3), which is consistent with reported studies that showed reduction in colony formation upon UPR induction (49, 50). The activation of caspases, a class of cysteine proteinases, is routinely used by us and others as indicators for the induction of apoptosis (40, 51, 52, 53, 54, 55, 56). The results show that SpiD7 activates effector caspases 3/7 in HGSC cell lines (CaOV3, OVCAR8, and Kuramochi) (Fig. 5*C*).

## Discussion

Here, we demonstrate that covalent modification of SEC residues induces UPR activation resulting in cancer cell death. In a previous study, we found that an isatin-derived spirocyclic core with an α-methylene-γ-butyrolactone moiety (analog **19**) inhibits NF-κB pathway by covalently binding to cysteine residues on RELA and IKKβ. Proteome-wide profiling of analog **19** targets using a click-pull down-MS study showed that analog **19** covalently modifies >330 proteins with high confidence. We also found that the modified proteins included those involved in key ER functions such as protein folding and cellular response to stress, thus indicating that **19** could be modulating the UPR.

We used TPE-MI to show elevated levels of SECs in cancer cells when compared with nontransformed immortalized cells indicating the presence of higher levels of unfolded proteins. This suggests that covalent modification of SECs will simulate the presence of misfolded proteins and disproportionately affect the cancer cells. Cells treated with UPR activator tunicamycin, which irreversibly binds to UDP-*N*-acetylglucosamine–dolichyl-phosphate *N*-acetylglucosamine-1-phosphate transferase, exhibit accumulation of unfolded/misfolded proteins in the ER and UPR activation (57). Treatment with lower dosage or shorter time results in adaptation or a lag period, whereas higher dosage and longer incubation times results in cell cycle arrest and eventually apoptosis by robust upregulation of CHOP and Gadd34 (58, 59).

Since **19** covalently modifies >330 proteins, we hypothesized that dimers that can covalently modify more than one sulfhydryl group and as a consequence simulate increased levels of misfolded proteins will activate UPR. The limited structure–activity relationship study with **19**, SpiD7, SpiD12, SpiD7-A, SpiD7-R, and SpiD7-C, identified structural element of SpiD7, such as linker length, the spirocyclic core, and the Michael acceptor, is required for activation of UPR. We recently showed that SpiD7 covalently modifies RELA to generate stable high molecular weight species (41). A logical extension is that SpiD7 simulates the presence of misfolded proteins to activate UPR. A comparison of SpiD7 activity in cancer cells (OVCAR5) *versus* normal cells (FT282C11) shows selective activation of IRE1 pathway in FT282C11 cells, indicating adaptation. It is important to note that when compared with FT282C11 cells, the basal XBP1 levels in OVCAR5 are elevated, which is probably required to handle the sustained cell intrinsic stress. This suggests that the IRE1 pathway in OVCAR5 cells is operating at maximal efficiency in its resting state. Activation of the PERK and ATF6 arms of the UPR pathway was observed in both OVCAR5 and FT282C11 cell lines. Analyses of time-course RNA-Seq datasets of OVCAR5 cells treated with SpiD7 identified 136 and 31 genes with *p* value <0.05 that were fourfold up and fourfold down, respectively, and exhibited a time-dependent increase or decrease (Table S3). Among the most differentially regulated genes, 99 mapped to an entity in reactome. Several stress response pathways including UPR activation were featured in the 20 most significant pathways that were perturbed by SpiD7 (Table S4).

In SV40-transformed mouse fibroblast, tunicamycin treatment selectively induced apoptosis in the transformed fibroblast by increasing the intracellular Ca^2+^ and accumulation of unglycosylated proteins in the ER (60). This is consistent with our observation of robust increase in cleaved-PARP and cleaved-caspase 7 levels in OVCAR5 cells treated with SpiD7 but not in FT282C11 cells. This suggests that IRE1 activation and robust XBP1 splicing observed in FT282C11 cells counteracts SpiD7-induced stress to block UPR-mediated apoptosis. Whereas in the cancer cells, the IRE1 pathway is activated in its resting state; therefore, there was no additional increase in XBP1 splicing upon SpiD7 treatment. XBP1 splicing is known to mitigate the ER stress in secretory B cells in multiple myeloma and triple-negative breast cancer cells to facilitate growth and survival (61, 62). This is similar to what we see in our study with FT282C11 cells wherein covalent modification of proteins by SpiD7 induces ER stress resulting in robust XBP1 splicing. Since IRE1 pathway is activated in resting OVCAR5 cells, the adaption threshold is breached upon SpiD7 treatment to activate programmed cell death. On the other hand, analog **19** did not induce the activation of UPR, indicating that at equimolar concentrations, dimer SpiD7–induced covalent modification of proteins is more effective in inducing UPR activation.

The time-course study with SpiD7 showed elevated levels of ER chaperone BiP as well as increase in p-eIF2α indicating activation of PERK, along with increased CHOP and cleaved-PARP protein levels. These observations are consistent with the RNA-Seq data that showed increased HSPA5 (BiP), EIF2AK3 (PERK), and DNA damage–inducible transcript 3 (DDIT3) (CHOP) levels. Previous studies with Tg, which is known to covalently modify the sarcoendoplasmic reticulum calcium transport ATPase pump, showed that disruption of the cellular Ca^2+^ homeostasis results in the induction of apoptosis by sustained elevation of ATF4, CHOP, and BiP followed by a gradual increase in cleaved PARP levels (63). Although SpiD7 and Tg activate UPR, our head-to-head comparison study of SpiD7 and Tg in normal *versus* cancer cells suggests that the mechanism associated with UPR activation plays a critical role in selectively inducing apoptosis in cancer cells. Growth inhibition studies showed that SpiD7 is approximately sixfold more potent inhibiting the growth of HGSC cell lines when compared with **19**. Moreover, depending on the HGSC cell lines, SpiD7 exhibited ∼3-fold to 15-fold selectivity in inhibiting the growth of HGSC cell line over FT282C11. Since SpiD7 also perturbs the NF-κB pathway, the observed inhibition of growth and induction of apoptosis is not exclusively because of UPR activation. Our studies provide critical proof of concept for a novel therapeutic modality; we recognize that additional studies with other dimers that have head groups that can covalently modify SECs are required to validate this strategy. In conclusion, our studies show that small molecules that possess the ability to covalently modify multiple SECs in protein complexes can selectively induce apoptosis in cancer cells by UPR activation.

## Experimental procedures

### Cell lines

Nontransformed human telomerase reverse transcriptase–immortalized human fallopian tube epithelial cells FT282C11 and human cancer cell lines MiaPaCa2, OVCAR5, OVCAR8, and CaOV3 were cultured in Dulbecco's modified Eagle's medium with high glucose (catalog no.: SH30022; Hyclone) with 10% fetal bovine serum (catalog no.: 26140079; Life Technologies) and 1% penicillin–streptomycin (catalog no.: 16777-164; Hyclone). Human cancer cell lines Kuramochi, SNU-119, and OVSAHO were cultured in RPMI1640 (catalog no.: SH30027; Hyclone) with 10% fetal bovine serum and 1% penicillin–streptomycin.

### Cell lysis and Western blot

Cell lysis and Western blot analyses were done following reported methods (52, 54, 55, 64, 65).

### Antibodies

ATF6 (CST; catalog no.: 65880), α-tubulin (CST; catalog no.: 3873), Cl-PARP (CST; catalog no.: 9541), PARP (CST; catalog no.: 9542), XBP1 (Abcam; catalog no.: Ab198999), p^S51^-eIF2α (CST; catalog no.: 3398), eIF2α (CST; catalog no.: 5324), Cl-Caspase-7 (CST; catalog no.: 8438), Bip (CST; catalog no.: 3177), PERK (CST; catalog no.: 5683), CHOP (CST; catalog no.: 2895), Caspase 7 (CST; catalog no.: 9492S), Caspase 9 (CST; catalog no.: 9502S), and XBP1 (Invitrogen; catalog no.: PA5-27650).

### RNA extraction and RNA-Seq analyses

OVCAR5 cells were seeded at a density of 2 × 10^6^ cells in 100 mm dishes and allowed to adhere overnight. The cells were treated with 10 μM of SpiD7 for 2, 6, and 12 h. The cells were washed with PBS post-treatment and incubated for 5 min at room temperature on shaker with 2 ml of Trizol (catalog no.: 15596018; Invitrogen). The samples were harvested and collected in microfuge tubes (1 ml per 2 ml tube). RNA purification steps were performed using the Direct-zol Miniprep Plus kit (catalog no.: #R2070; Zymo Research) according to the manufacturer’s protocol.

RNA-Seq of the samples was performed at the UNMC Genomics Core Facility following reported method (66) using two lanes of the HiSeq 2500 DNA Analyzer (Illumina) to generate a total of approximately 20 to 25 million 50 bp single reads for each sample. The quality of the sequencing was continually monitored with a Q30 score. Following sequencing, the samples were demultiplexed to produce FASTQ files. The resulting sequence files were processed by the UNMC Epigenomics core facility. Adaptor sequences and low-quality (Phred score: 20) ends were trimmed using the Trim Galore software package (http://www.bioinformatics.babraham.ac.uk/projects/trim_galore/). The resulting FASTQ files were aligned to the human genome (GRCm38/mm10) using the software TopHat (version 2.0.8) (http://ccb.jhu.edu/sofware/tophat/index.shtml). The software Cufflinks (version 2.1.1) (http://cole-trapnell-lab.github.io/cufflinks/) was used to estimate the expression values, and Cuffdiff (version 2.1.1; cufflinks) was used to determine the differential expression.

### Colony formation assay

Colony formation assay was done following reported methods (67). Briefly, single-cell suspension of OVCAR8 and Caov3 cells was seeded in 6-well dishes at a density of 1000 cells per well in triplicates. After overnight incubation, cells were treated with different concentrations of SpiD7 and allowed to form colonies for 7 or 14 days. After incubation, cells were fixed with methanol, stained with 0.5% crystal violet in PBS, rinsed with water, and air dried overnight. Colonies containing >50 cells were counted using inverted light microscope manually.

### Growth inhibition and caspase 3/7 assays

Growth inhibition and caspase 3/7 assay was done following reported methods (46, 51, 53, 54, 55, 68, 69, 70, 71).

### Statistical methods

The mean ± SD of biological replicates was used to generate the graphs, and statistical analyses were performed using two-tailed Student’s *t* test.

## Data availability

All data generated and analyzed in this study are included in the article or can be obtained from the authors upon reasonable request. The MS proteomics data have been deposited to the ProteomeXchange Consortium *via* the PRIDE (72) partner repository with the dataset identifier PXD029783 and 10.6019/PXD029783. All FASTQ files were deposited in the Gene Expression Omnibus database under accession number GSE190368. Please direct all requests to Amarnath Natarajan (anatarajan@unmc.edu).

## Supporting information

This article contains supporting information (40, 41, 73, 74, 75).

## Conflict of interest

A. N. and S.R. are listed as inventors on US patent 11,104,684. All other authors declare that they have no conflicts of interest with the contents of this article.
