# Supplementary material for: Spirocyclic dimer SpiD7 activates the unfolded protein response to selectively inhibit growth and induce apoptosis of cancer cells
Source: J Biol Chem. 2022 Apr 1;298(5):101890. doi: 10.1016/j.jbc.2022.101890 (PMC9062249; doi:10.1016/j.jbc.2022.101890)
Supplement: Supplemental Figures S1–S4 and Tables S2 and S4 [file mmc1.pdf]

Supporting information

Spirocyclic dimer SpiD7 activates the unfolded protein response to selectively inhibit growth and induce apoptosis of cancer cells

Smit Kour,[a‡] Sandeep Rana,[a‡] Sydney P. Kubica,[a] Smitha Kizhake,[a] Mudassier Ahmad,[a] Catalina Muñoz-Trujillo,[a] David Klinkebiel,[a] Sarbjit Singh,[a] Jayapal Reddy Mallareddy,[a] Surabhi Chandra,[b] Nicholas T. Woods,[a] Adam R. Karpf,[a] and Amarnath Natarajan.[a*]

Chemistry Experimental: All reagents were purchased from commercial sources and were used without further purification. Flash chromatography was carried out on silica gel (200–400 mesh). Thin layer chromatography (TLC) was run on pre-coated EMD silica gel 60 F254 plates and observed under UV light at 254 nm and with basic potassium permanganate dip. Column chromatography was performed with silica gel (230-400 mesh, grade 60, Fisher scientific, USA). ^{1}H NMR and ^{13}C NMR spectra were recorded in chloroform-d_3 or DMSO-d_6 on a Varian-500, Varian-600 and Bruker-500 spectrometer (DMSO-d_6 2.50 ppm for ^{1}H and 39.00 ppm for ^{13}C and $CDCl_3$ was 7.26 ppm for ^{1}H and 77.00 ppm for ^{13}C. Proton and carbon chemical shifts were reported in ppm relative to the signal from residual solvent proton and carbon. The purity of all final compounds was ≥95% as determined by analytical HPLC on a reverse-phase column (Zorbax 300SB C18, 2.1 × 150 mm, 5 μm particle size) using an Agilent 1200 series system with UV detector (214nm and 254nm) with the binary system water/acetonitrile containing 0.1% trifluoracetic acid (TFA) as eluent or Analytical HPLC was carried out on 250 x 4.60 mm C-18 column using gradient conditions (10 – 100% B, flow rate = 1.0 mL/min, 15 min). The eluents used for purification were solvent A (H_2O with 0.1% Formic acid) and solvent B (CH_3CN with 0.1% Formic acid).

Synthesis of analog 19: Analog 19 was synthesized by using the procedure reported by us previously.(1)

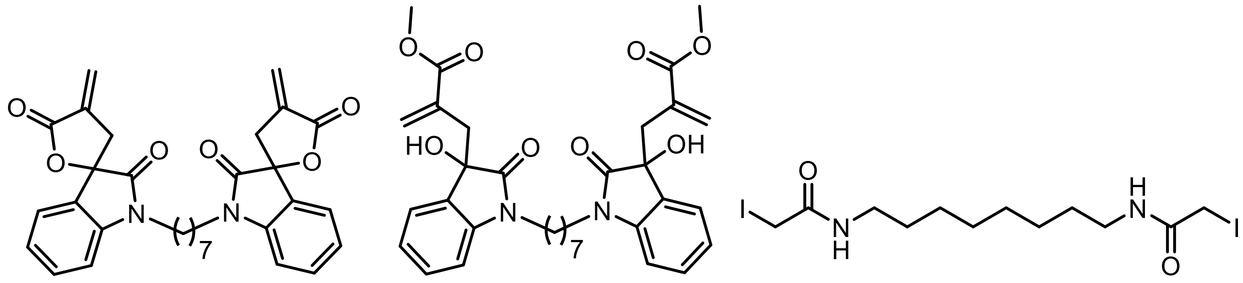

Synthesis of SpiD7, SpiD7-A and SpiD7-C: The cyclic (SpiD7), acyclic SpiD7 (SpiD7-A) and the control (SpiD7-C) was synthesized by using the procedure reported by us previously.(2)

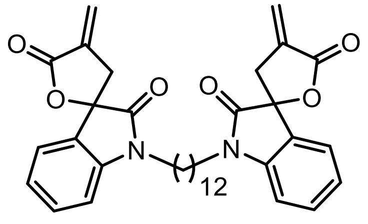

Synthesis of SpiD12: SpiD12 was synthesized by using the same procedure as used for SpiD7 using a 12-carbon linker. ^{1}H NMR (499 MHz, $CDCl_3$) δ 7.38 (t, *J* = 7.8 Hz, 2H), 7.31 (d, *J* = 7.5 Hz, 2H), 7.10 (t, *J* = 7.6 Hz, 2H), 6.88 (d, *J* = 7.9 Hz, 2H), 6.41 – 6.40 (m, 2H), 5.80 – 5.79 (m, 2H), 3.72 – 3.62 (m, 4H), 3.31 – 3.07 (m, 4H), 1.70 – 1.65 (m, 4H), 1.32 – 1.25 (m, 16H). ^{13}C NMR (126 MHz, $CDCl_3$) δ 173.36, 169.01, 143.40, 132.87, 131.19, 126.85, 124.27, 123.38, 123.01, 109.22, 79.29, 40.34, 36.32, 29.41, 29.35, 29.14, 27.16, 26.76. HRMS (ESI-MS) calcd for $C_{36}H_{41}N_2O_6$ $[M+H]^+$: 597.2959; found 597.2961.

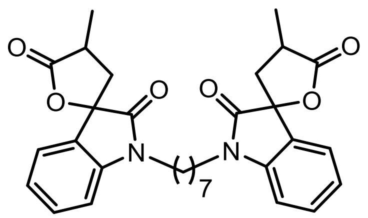

Synthesis of SpiD7-R: To a solution of SpiD7 (1 eq) in a round bottom flask in dry THF was added Pd/C (0.2 eq, 5 % by weight on activated carbon). The reaction was vacuumed and purged

with hydrogen three times followed by additional stirring under hydrogen atmosphere for 5 hours. The progress of the reaction was monitored by TLC. After completion of reaction, the reaction mixture was passed through a bed of celite and column chromatographed using hexane and ethyl acetate gradient to obtain the desired product. ^{1}H NMR (499 MHz, $CDCl_3$) δ 7.39 – 7.33 (m, 4H), 7.13 – 7.07 (m, 2H), 6.87 – 6.84 (m, 2H), 3.67 – 3.59 (m, 4H), 3.51 – 3.43 (m, 2H), 2.70 – 2.65 (m, 2H), 2.13 (t, *J* = 2.5 Hz, 2H), 1.66 – 1.65 (m, 4H), 1.51 – 1.49 (m, 2H), 1.41 – 1.30 (m, 10H). ^{13}C NMR (126 MHz, $CDCl_3$) δ 178.82, 178.50, 174.29, 143.45, 142.88, 131.10, 130.91, 127.91, 126.30, 124.46, 123.77, 123.33, 123.27, 109.24, 109.05, 80.53, 80.20, 40.25, 39.98, 39.84, 38.36, 34.72, 34.33, 28.72, 28.70, 27.04, 26.55, 16.41, 15.50. ESI-MS calcd for $C_{31}H_{34}N_2O_6$ $[M]^+$: 530.24; found 553.64 (M + Na).

Synthesis of TPE-MI dye: The TPE-MI dye was synthesized by using the procedure reported by Liu et al. except a following change in the purification method.(3) The TPE-MI was purified by column chromatography using ethyl acetate and hexane (2:8) as eluting solvents; Yield = 53%.

Click chemistry: MiaPaCa2 cells were allowed to grow to 90% confluency in a 10 cm flask under normoxic conditions at 37 °C in a humidified CO_2 incubator. The cells were treated with 10 μM of Analog **20** for 1h. After incubation, the medium was removed, and the cells were washed with cold PBS. The cells were lysed in a buffer comprised of Radioimmunoprecipitation assay (RIPA) buffer (Thermo Scientific), sodium orthovandate (Na_3VO_4, Sigma), sodium flouride (NaF, Sigma), β-glycerophosphate (Sigma) and 1mM phenylmethylsulfonyl fluoride (PMSF, Sigma). The samples were incubated on ice for 30 minutes and vortexed at 15-minute intervals. The lysates were centrifuged at 14,000 g for 10 min and supernatant was collected. The lysates were stored at −80 °C until further use. Click chemistry reaction was performed using 1mg of the thawed lysate. Click reagents (10 µL copper sulphate, 50 mM stock in ddH2O; 15 µL tris[1-benzyl-1H-1,2,3-triazol-4-yl)methyl]amine TBTA, 1.35 mM stock in DMSO; 10 µL TAMRA-Biotin-azide [Sigma-Aldrich #762024], 1 mM stock in DMSO; 10 µL tris(2- carboxyethyl) phosphine hydrochloride TCEP, 50 mM in ddH2O) were added and the reaction was allowed to proceed for 4 h at RT in dark. The sample was washed 3x with 10 mL methanol to pellet the precipitated proteins and air dried. Next, we added 0.2% SDS in PBS and 100 μL high-capacity streptavidin agarose resin (Thermo Scientific, #20361) and incubated at 4 ºC for 72h. The sample was kept at RT for 3 h and washed twice with PBS and water. Next, we added 600µL of 6 M urea/PBS to make total volume up to 1 mL, followed by adding 50 µL DTT (200mM stock in water) and heated at 65 ºC for 15min. This was followed by addition of Iodoacetamide (final conc. 15mM,

stock 0.5M) and was kept at RT for 30 mins in dark. The beads were pelleted and resuspended in 50 µL of 6 M urea/PBS. Sequencing grade modified trypsin (20 µg dissolved in 100 µL of trypsin resuspension buffer, Promega) was added and digested overnight at 37 °C. The digested sample was separated from the beads at 1.7 rcf for 5 min using a Micro Bio-Spin column (BioRad). The beads were washed twice with 50 µL water and the washes were combined with the eluted peptides and submitted for MS.

LC-MS/MS and database searches: Peptides generated by typsin digest were analyzed by LC-MS/MS on an RSLCnano system (ThermoFisher Scientific) coupled to a Q-Exactive HF mass spectrometer (ThermoFisher Scientific). The samples were first injected onto a trap column (Acclaim PepMap™ 100, 300 µm x 0.5 cm, ThermoFisher Scientific) for 3.0 min at a flow rate of 5 µL/min before switching in line with the main column. Separation was performed on a C18 nano column (Acclaim PepMap™ 100, 2µm 75µm x 250mm, ThermoFisher Scientific) at 260 nL/min with a linear gradient from 5-32% over 36 min. The LC aqueous mobile phase contained 0.1% (v/v) formic acid in water and the organic mobile phase contained 0.1% (v/v) formic acid in 80% (v/v) acetonitrile. Mass spectra for the eluted peptides were acquired on a Q Exactive HF mass spectrometer in data-dependent mode using a mass range of m/z 250–1500, resolution 120,000, AGC target 3 x 10^6, maximum injection time 60 ms for the MS1 peptide measurements. Data-dependent MS2 spectra were acquired by HCD as a Top12 experiment with a normalized collision energy (NCE) set at 28%, AGC target set to 1 x 10^5, 15,000 resolution, intensity threshold 2 x 10^4 and a maximum injection time of 250 ms. Dynamic exclusion was set at 12 sec and the isolation window set to 1.6 *m/z***.**

Database searching: The mass spectra were extracted using Proteome Discoverer Daemon (2.4.1.15). All MS/MS samples were analyzed using Mascot (Matrix Science, London, UK; version 2.5.1). Mascot was set up to search the cRAP_20150130 database (selected for Homo sapiens, unknown version, 20295 entries) assuming the digestion enzyme trypsin allowing for up to 3 missed protease cleavage sites. Mascot was searched with a fragment ion mass tolerance of 0.060 Da and a parent ion tolerance of up to 15.0 PPM. O+14 of pyrrolysine was specified in Mascot as a fixed modification. Deamidated of asparagine and glutamine, oxidation

of methionine and carbamidomethyl of cysteine were specified in Mascot as variable modifications. False discovery rates were determined searching the decoyed database.

Criteria for protein identification: Scaffold (version Scaffold_4.10.0, Proteome Software Inc., Portland, OR) was used to validate MS/MS based peptide and protein identifications. Peptide identifications were accepted if they could be established at greater than 95.0% probability by the Peptide Prophet algorithm (4) with Scaffold delta-mass correction. Protein identifications were accepted if they could be established at greater than 95.0% probability and contained at least 1 identified peptide. Protein probabilities were assigned by the Protein Prophet algorithm (5). Proteins that contained similar peptides and could not be differentiated based on MS/MS analysis alone were grouped to satisfy the principles of parsimony. Proteins sharing significant peptide evidence were grouped into clusters.

Figure S1

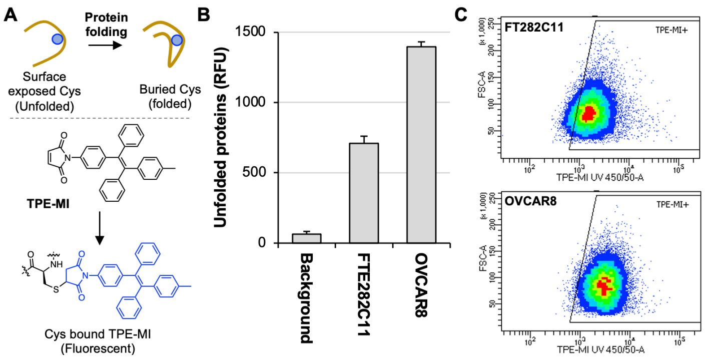

(**A**) Cartoon shows that there is more surface exposed Cys residues in unfolded proteins and TPE-MI dye becomes fluorescent when it covalently binds to unfolded proteins. (**B**) Levels of unfolded proteins in immortalized fallopian tube epithelial cells (FT282C11) and HGSC cells (OVCAR8) (**C**) Flow cytometry study with TPE-MI treated FT282C11 and OVCAR8 cells.

Figure S2

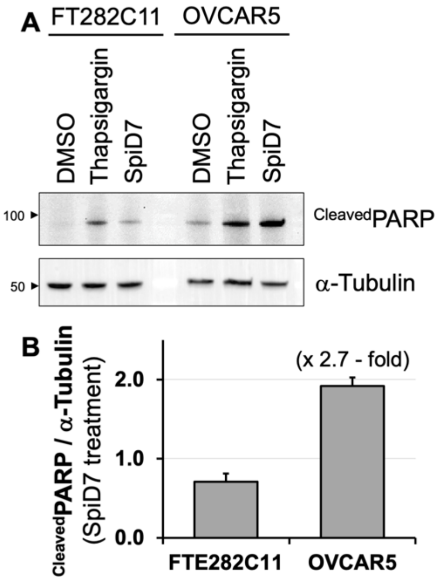

(**A**) FT282C11 and OVCAR5 cells were treated with 10μM thapsigargin (Tg) or dimer SpiD7 and incubated for 6h. The lysates were subjected to Western blot analyses and the membranes probed for ^{cleaved}PARP, and α-Tubulin. (**B**) SpiD7 treatment results is ~2.7-fold higher levels of ^{cleaved}PARP in OVCAR5 cells.

Figure S3

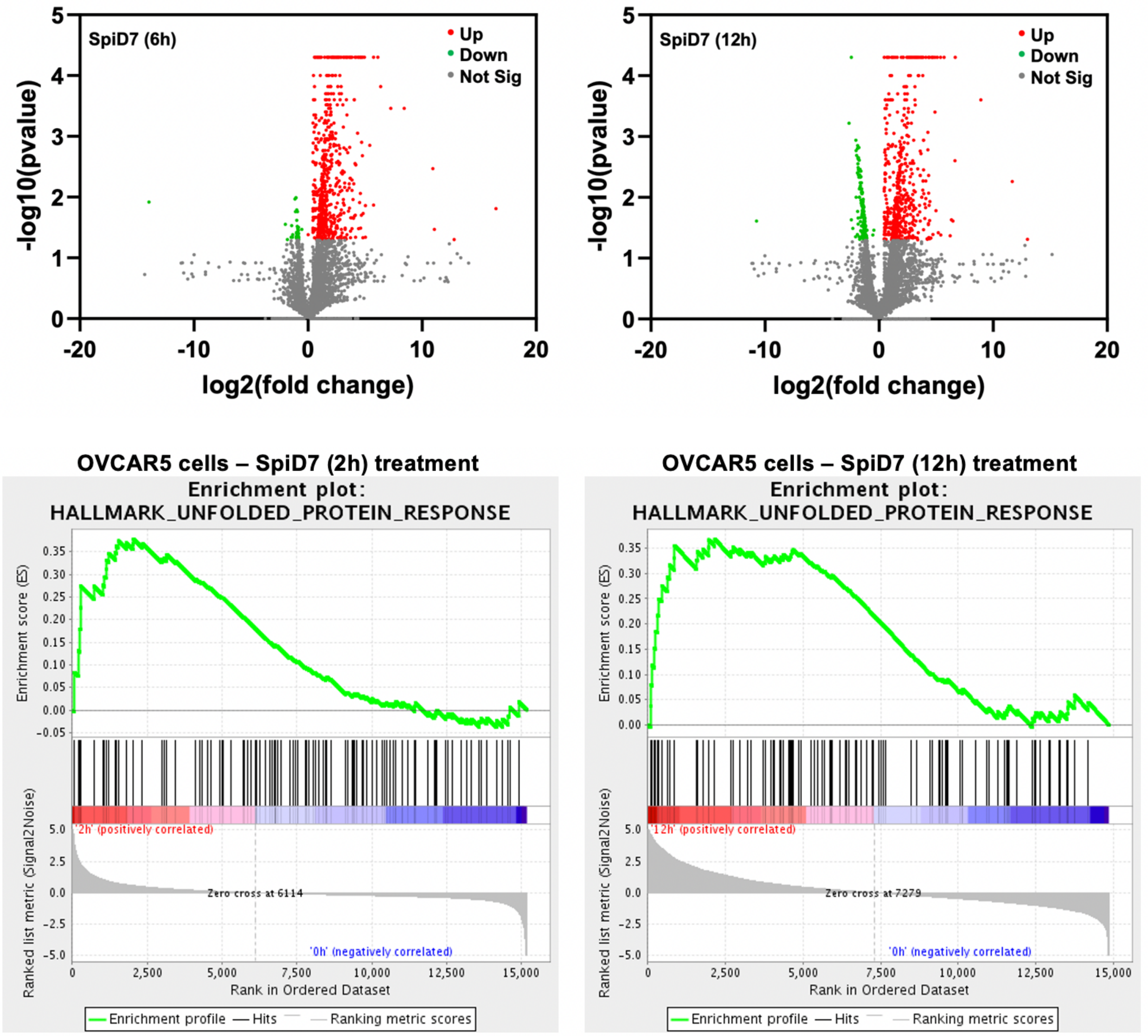

(**Top**) Volcano plot showing the differential RNA expression between the 6h and 12h vehicle and SpiD7 treatment against p value (≤0.05 and fold 2-fold change is highlighted in red and green). (**Bottom**) Gene Set Enrichment Analysis (GSEA) for the Hallmark UPR gene set for 2h and 12h, SpiD7 treated cells (FDR ≤0.05).

Figure S4

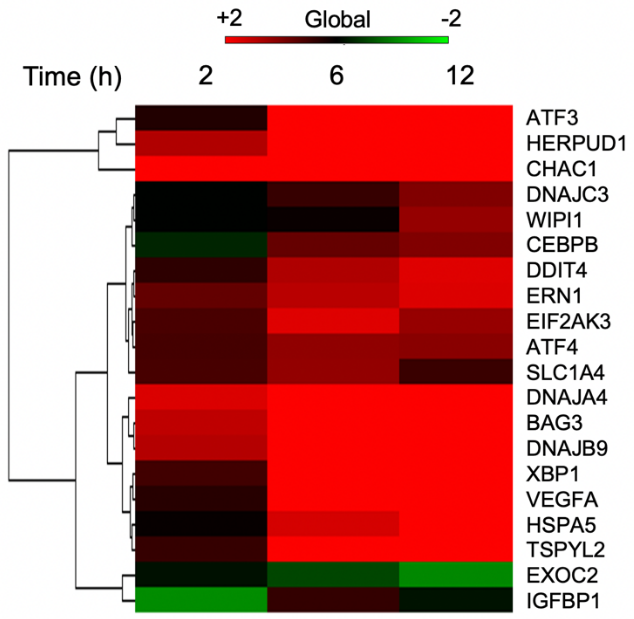

Heat map showing 2-fold change of the Hallmark UPR gene set for the 2, 6, and 12 h, SpiD7 treated cells.

Table S1: Click-MS dataset (see attached excel sheet).

Table S2: The top 20 most significant pathways sorted by p-value for the Click-MS dataset.

Pathway name	Entities				Reactions	
	found	ratio	p-value	FDR*	found	ratio
Peptide chain elongation	52 / 97	0.006	1.11e-16	3.22e-15	5 / 5	3.66e-04
Eukaryotic Translation Elongation	53 / 102	0.007	1.11e-16	3.22e-15	9 / 9	6.60e-04
Nonsense Mediated Decay (NMD) independent of the Exon Junction Complex (EJC)	52 / 101	0.007	1.11e-16	3.22e-15	1 / 1	7.33e-05
Formation of a pool of free 40S subunits	54 / 106	0.007	1.11e-16	3.22e-15	2 / 2	1.47e-04
GTP hydrolysis and joining of the 60S ribosomal subunit	59 / 120	0.008	1.11e-16	3.22e-15	3 / 3	2.20e-04
L13a-mediated translational silencing of Ceruloplasmin expression	58 / 120	0.008	1.11e-16	3.22e-15	3 / 3	2.20e-04
Formation of the ternary complex, and subsequently, the 43S complex	24 / 54	0.004	1.11e-16	3.22e-15	3 / 3	2.20e-04
Nonsense-Mediated Decay (NMD)	55 / 124	0.008	1.11e-16	3.22e-15	6 / 6	4.40e-04
Nonsense Mediated Decay (NMD) enhanced by the Exon Junction Complex (EJC)	55 / 124	0.008	1.11e-16	3.22e-15	5 / 5	3.66e-04
Viral mRNA Translation	50 / 114	0.008	1.11e-16	3.22e-15	2 / 2	1.47e-04
SRP-dependent cotranslational protein targeting to membrane	51 / 119	0.008	1.11e-16	3.22e-15	5 / 5	3.66e-04
Translation initiation complex formation	26 / 62	0.004	1.11e-16	3.22e-15	2 / 2	1.47e-04
Ribosomal scanning and start codon recognition	26 / 64	0.004	1.11e-16	3.22e-15	2 / 2	1.47e-04
Major pathway of rRNA processing in the nucleolus and cytosol	54 / 189	0.013	1.11e-16	3.22e-15	7 / 7	5.13e-04
Eukaryotic Translation Initiation	59 / 130	0.009	1.11e-16	3.22e-15	20 / 21	0.002
Cap-dependent Translation Initiation	59 / 130	0.009	1.11e-16	3.22e-15	17 / 18	0.001
Activation of the mRNA upon binding of the cap-binding complex and eIFs, and subsequent binding to 43S	26 / 66	0.004	1.11e-16	3.22e-15	5 / 6	4.40e-04
Eukaryotic Translation Termination	51 / 106	0.007	1.11e-16	3.22e-15	4 / 5	3.66e-04
Translation	84 / 339	0.022	1.11e-16	3.22e-15	66 / 99	0.007
rRNA processing in the nucleus and cytosol	54 / 208	0.014	1.11e-16	3.22e-15	8 / 15	0.001

*False Discovery Rate

Table S3: Genes that were up or down regulated 4-fold with p-value < 0.05 that were subjected to Euclidean hierarchical clustering (please see attached excel file)

Table S4: The top 20 most significant pathways sorted by p-value for genes that were up or down regulated 4-fold with p-value < 0.05 in RNA-seq datasets.

Pathway name	Entities				Reactions	
	found	ratio	p-value	FDR*	found	ratio
Attenuation phase	19 / 47	0.003	1.11e-16	1.42e-14	3 / 5	3.66e-04
HSF1-dependent transactivation	20 / 59	0.004	1.11e-16	1.42e-14	4 / 8	5.86e-04
Regulation of HSF1-mediated heat shock response	24 / 113	0.007	1.11e-16	1.42e-14	7 / 14	0.001
Cellular response to heat stress	25 / 135	0.009	1.11e-16	1.42e-14	12 / 29	0.002
HSF1 activation	16 / 43	0.003	1.11e-16	1.42e-14	1 / 7	5.13e-04
Cellular responses to stress	45 / 953	0.063	1.65e-12	1.77e-10	46 / 381	0.028
Cellular responses to stimuli	45 / 971	0.064	3.10e-12	2.82e-10	46 / 412	0.03
Response of EIF2AK1 (HRI) to heme deficiency	5 / 29	0.002	7.20e-05	0.006	6 / 20	0.001
TP53 Regulates Transcription of Genes Involved in G2 Cell Cycle Arrest	4 / 21	0.001	2.67e-04	0.019	6 / 11	8.06e-04
RAF-independent MAPK1/3 activation	4 / 28	0.002	7.82e-04	0.05	4 / 12	8.79e-04
MAPK3 (ERK1) activation	3 / 13	8.63e-04	9.39e-04	0.054	2 / 4	2.93e-04
NGF-stimulated transcription	5 / 56	0.004	0.001	0.075	7 / 37	0.003
Interleukin-33 signaling	2 / 4	2.65e-04	0.002	0.078	2 / 2	1.47e-04
Unfolded Protein Response (UPR)	8 / 155	0.01	0.002	0.091	7 / 94	0.007
G0 and Early G1	4 / 38	0.003	0.002	0.1	4 / 27	0.002
TP53 Regulates Transcription of Cell Cycle Genes	5 / 65	0.004	0.003	0.102	7 / 42	0.003
Cell-extracellular matrix interactions	3 / 19	0.001	0.003	0.102	3 / 10	7.33e-04
Formation of the beta-catenin:TCF transactivating complex	5 / 67	0.004	0.003	0.107	4 / 13	9.53e-04
Transcriptional regulation of granulopoiesis	5 / 71	0.005	0.004	0.129	2 / 27	0.002
Nuclear Events (kinase and transcription factor activation)	5 / 80	0.005	0.006	0.204	7 / 48	0.004

*False Discovery Rate

References

1. Rana, S., Blowers, E. C., Tebbe, C., Contreras, J. I., Radhakrishnan, P., Kizhake, S., Zhou, T., Rajule, R. N., Arnst, J. L., Munkarah, A. R., Rattan, R., and Natarajan, A. (2016) Isatin Derived Spirocyclic Analogues with alpha-Methylene-gamma-butyrolactone as Anticancer Agents: A Structure-Activity Relationship Study. *J Med Chem* **59**, 5121-5127
2. Kour, S., Rana, S., Kizhake, S., Lagundzin, D., Klinkebiel, D., Mallareddy, J. R., Huxford, T., Woods, N. T., and Natarajan, A. (2022) Stapling proteins in the RELA complex inhibits TNFalpha-induced nuclear translocation of RELA. *RSC Chem Biol* **3**, 32-36
3. Liu, Y., Yu, Y., Lam, J. W., Hong, Y., Faisal, M., Yuan, W. Z., and Tang, B. Z. (2010) Simple biosensor with high selectivity and sensitivity: thiol-specific biomolecular probing and intracellular imaging by AIE fluorogen on a TLC plate through a thiol-ene click mechanism. *Chemistry* **16**, 8433-8438
4. Keller, A., Nesvizhskii, A. I., Kolker, E., and Aebersold, R. (2002) Empirical statistical model to estimate the accuracy of peptide identifications made by MS/MS and database search. *Anal Chem* **74**, 5383-5392
5. Nesvizhskii, A. I., Keller, A., Kolker, E., and Aebersold, R. (2003) A statistical model for identifying proteins by tandem mass spectrometry. *Anal Chem* **75**, 4646-4658
